# Improved Soil Amendment by Integrating Metal Complexes and Biodegradable Complexing Agents in Superabsorbents

**DOI:** 10.3390/ma17010141

**Published:** 2023-12-27

**Authors:** Alicja Drozd, Yongming Ju, Dorota Kołodyńska

**Affiliations:** 1 Analytical Department, Łukasiewicz Research Network—New Chemical Syntheses Institute, Al. Tysiąclecia Państwa Polskiego 13a, 24-110 Puławy, Poland; alicja.drozd@ins.lukasiewicz.gov.pl; 2 Nanjing Institute of Environmental Sciences, Ministry of Ecology and Environment (MEE), Nanjing 510655, China; juyongming@scies.org; 3 Innovative Laboratory for Environmental Functional Materials and Environmental Applications of Microwave Irradiation, South China Subcenter of State Environmental Dioxin Monitoring Center, South China Institute of Environmental Sciences, Ministry of Ecology and Environment (MEE), Guangzhou 510655, China; 4 Faculty of Chemistry, Institute of Chemical Science, Department of Inorganic Chemistry, Maria Curie-Sklodowska University, M. Curie-Sklodowska Sq. 2, 20-031 Lublin, Poland

**Keywords:** complexing agents, IDHA, EDDS, GLDA: fertilizers, sorption assessment

## Abstract

The superabsorbents’ application as materials for the preparation of modern mineral fertilizers of controlled activity is presented. Under the static conditions, the commercial acrylic-based Agro^®^ Hydrogel was used as a sorbent for Cu(II), Fe(III), Mn(II), and Zn(II) ions in the presence of three biodegradable complexing agents of the new generation: (N-1,2-dicarboxyethyl)-D,L-aspartate acid (IDHA), N,N-ethylenediaminedisuccinic acid (EDDS) and N,N-bis(carboxymethyl) glutamic acid (GLDA). The ions and complexes concentrations were determined by the inductively coupled plasma optical emission spectrometer (ICP-OES). The characterization of hydrogel before and after the adsorption process was made using the Fourier transform infrared spectroscopy (FT-IR), surface area determination (ASAP), scanning electron microscopy (SEM-EDS) as well as the thermogravimetric (TGA) methods. The influence of the phase contact time, initial concentration, and pH on the adsorption capacities was investigated. The kinetic and adsorption parameters were determined. The Langmuir, Freundlich, Dubinin–Radushkevich, and Temkin adsorption models were applied to describe the experimental data. The Langmuir isotherm model accurately characterized the equilibrium process. The adsorption process was fast, and it reached equilibrium after 60 min of the phase contact time. The research on the adsorption of Cu(II), Fe(III), Mn(II), and Zn(II) onto Agro^®^ Hydrogel with IDHA, EDDS, and GLDA indicates that these complexing agents improve process efficiency.

## 1. Introduction

Currently, agriculture is one of the most important sectors of the economy in many countries [1,2,3]. In order to intensify agricultural production, an increase in the consumption of mineral fertilizers is observed, so new technologies for fertilizer manufacture are being worked out [4,5,6]. Over 200 manufacturers of fertilizers and soil conditioners are active on the national market. The essential factor determining the choice of technologies and preparations to utilize in the cultivation of plants, is their influence on the environment. Through many years of reckless practices (over-fertilization, irrigation, and intensive use of chemical plant protection products), the natural environment is constantly destroyed. On the other hand, recent irregular rainfalls and long periods of drought resulted in small yields of field crops. Therefore, solutions to secure the maintenance of optimal physical properties of soil, mainly improvement of porosity, are searched for [7,8,9].

One such method is the use of polymer superabsorbents, commonly known as hydrogels. They are three-dimensional cross-linked hydrophilic polymers that can absorb and retain large volumes of water up to thousands of times their own weight, and the absorbed water is hardly removable, even under pressure. This phenomenon of water absorption is caused by the loosening of polymer networks, resulting in material swelling and assuming a gel form [10,11,12,13]. Hydrogels are classified on the basis of such parameters as physical properties, origin, cross-linking, preparation, degradability, and ionic charge. They possess very important features: large adsorption capacity, high rate of reversible fluid absorption, mechanical strength, non-toxicity, chemical resistance, as well as mechanical resistance and water absorption possibilities in the saline solution [14,15,16]. Physical and chemical properties make it so that for over fifty years, hydrogels have sparked great interest in numerous industries. They are commonly used in the production of drugs, medical dressings, cosmetics, means of personal hygiene, elements of surgical implants, heat-sensitive surfaces, tissue engineering, etc. [12,13,17,18,19,20,21]. Additionally, they can be used in agriculture and horticulture because they can help reduce irrigation water consumption and the death rate of plants, improve fertilizer retention in soil, and increase plant growth rate [22,23,24,25]. In addition to water absorption and retention, they have many advantages over con-ventional biochars, such as sustained supply of nutrients to plants for a longer period of time, thus increasing the phosphate fertilizer efficiency and decreasing the applica-tion frequency. Furthermore, they can be used as soil moisture preservation materials [26,27,28,29,30]. Hydrogels designed for agricultural applications are eco-friendly and, at the same time, allow us to limit the negative effects of drought and revitalize natural re-sources. Basic economic and social benefits: reducing the need for irrigation by about 20–50%, preventing the effects of water stress and drought, limiting fertilizer doses by approx. 30% through the ‘intelligent’ way of releasing nutrients within the root system, protecting the natural environment against the effects of over-fertilization and land salinity limiting the leaching of fertilizers into deeper soil parts, protecting groundwa-ter, limiting the intensity of care treatments by approx. 9%, and increasing yield by approx. 15%. As such they have the ability to modify the local microenvironment of seeds/seedlings to enhance their growth outcomes. The network structure of hydrogels is typically formed by physical interaction and/or chemical cross-linking between polymer chains. The nature, strength and extent of cross-linking can be tailored to customize gel properties (such as mechanical strength, porosity and swelling behavior) to suit a given type of application [3]. 

In the present study, another application of the commercial hydrogel Agro^®^ Hydrogel was presented. It was used as the prototype of the novel fertilizer containing micronutrients such as Cu(II), Zn(II), Mn(II), and Fe(III) in the form of complexes with iminodisuccinic acid (IDHA), N,N-ethylenedisuccinic acid (EDDS) or N,N-bis(carboxylmethyl)-L-glutamic acid (GLDA). These chelating agents belong to the class of a novel group of complex aminopolycarboxylic acids type, which are characterized by high biodegradability compared to the traditional chelating agents such as commonly applied EDTA (ethylenediaminetetraacetic acid). They are also non-toxic, biologically inert, and soluble in water, and their degradation does not result in environmental pollution [31,32,33]. Such compounds become available on the market due to legislative changes [34]. All the prominent chemical companies, Lanxess, Akzo Nobel, and BASF, have them in their sales portfolio. In accordance with Regulation (EC) No 2003/2003 of the European Parliament and the Council of 13 October 2003 (as modified) regarding fertilizer, only a small number of compounds have been utilized to this point. The most significant ones have been used, including ethylenediaminetetraacetic acid EDTA, N-(1,2-dicarboxyethyl)-D,L-aspartic acid–IDHA, ethylenediaminedisuccinic acid–EDDS, ethylenediamine-N-N-bis(o-hydroxyphenylacetic) acid–EDDHA as well as N,N-bis(2-hydroxy-5-methylbenzyl)ethylenediamine-N,N-diacetic acid–HJB and N,N-bis(2-hydroxybenzyl)ethylenediamine-N,N’-diacetic acid–HBED [34]. It is well-known that by combining micronutrients with complexing agents, fertilizers are less likely to react negatively with the soil water system. Plants can still have access to nutrients in the usable form of complex structures. The usage of slow-release fertilizer hydrogels, which combine a fertilizer with a hydrogel, reduces the frequency of irrigation and the loss of soil moisture through evaporation. Additionally, nearly more than half of the fertilizers and nutrients used to increase agricultural yields worldwide are wasted. Therefore, it is imperative to use workable, environmentally acceptable technology to increase the number of nutrients in soil that are available to plants. 

Taking these into account, the goal of this research is to determine the feasibility of using Agro® Hydrogel for Cu(II), Zn(II), Mn(II), and Fe(III) ion incorporation in the presence of the new generation chelating agents such as IDHA, EDDS, and GLDA. As presented in our previous paper, complex micronutrients were successfully incorporated utilizing a sorption approach [32]. The adsorption of the Cu(II), Zn(II), Mn(II), and Fe(III) complexes on Agro® Hydrogel was investigated as a function of sorbent dose, pH value, initial concentration, phase contact time, and temperature. Furthermore, to get to know the incorporation mechanism, there were also applied the models to fit the adsorption equilibrium and kinetic data: the pseudo-first-order (PFO), the pseudo-second-order (PSO), and the intraparticle diffusion (IPD) ones. From the linear dependence of the Langmuir, Freundlich, Dubinin–Raduszkiewicz, and Temkin isotherms, the maximal adsorption capacities and the constants for the studied Agro^®^ Hydrogel sorbent were determined. The physicochemical characterization of the used hydrogel was also presented.

## 2. Materials and Methods

### 2.1. Chemicals

Agro^®^ Hydrogel (AH) (EverChem, Łowicz, Poland) was chosen for study. It is characterized by a cross-linked polyacrylic backbone (acrylamide-polyacrylate), a large absorption capacity of distilled water (up to 380 g H_2_O per 1 g of hydrogel), and grain sizes of up to 1 mm. The optimal operating pH range for the selected hydrogels indicated by the manufacturers is 5–9 (Table S1). 

The metal ions Cu(II), Zn(II), Mn(II), and Fe(III) were chosen for the kinetic and adsorption studies (Avantor Performance Materials, Gliwice, Poland). Table S2 lists the concentrations of micronutrients used in the investigation and the abbreviations of the obtained complexes. The aqueous solutions of each metal ion in the presence of IDHA (Lanxess, Cologne, Germany), EDDS (Innospec Inc., Englewood, CO, USA), or GLDA (AkzoNobel, Amsterdam, The Netherlands) were prepared to dissolve the equimolar amounts of metal ions in the IDHA, EDDS or GLDA solutions. The other commercially available chemicals, e.g., NaOH, NaCl, and HCl of analytical reagent grade, were provided by Avantor Performance Materials, Poland.

### 2.2. Analytical Methods

The swellability of Agro^®^ Hydrogel was determined, allowing the sample to swell in water and NaCl. For studying the absorbency rate, 0.1 g ± 0.001 g, samples with various particle sizes were put into calibrated flasks and immersed in 50 mL distilled water. At appropriate time intervals, the water absorbency of the hydrogel was determined by weighing the swollen samples [35,36]. The procedure is presented in the Supplementary Materials section. The analogous procedure was applied for NaCl. The water retention capacity of tested hydrogel over time, Q_H2O_,% and Q_NaCl_, and % absorbencies of Agro^®^ Hydrogel were compared.

As for pH response performance tests of hydrogel buffer solutions 2, 4.01, 7, and 11 were used. Dry gel samples with a mass of 0.1 g ± 0.001 g with various particle sizes were put into calibrated flasks and immersed in 50 mL of buffers until the swelling was balanced. The equation for calculating the equilibrium swelling (Q_buffer_, %) rate was as previously presented in the function of pH.

The Fourier transform infrared (FTIR) spectra of Agro^®^ Hydrogel before and after incorporation of Cu(II), Zn(II), Mn(II), and Fe(III) complexes with IDHA, EDDS, and GLDA were recorded with an FTIR spectrometer Cary 630 (Agilent Technologies, Santa Clara, CA, USA) and the Agilent MicroLab FTIR software (Agilent Technologies, Santa Clara, CA, USA).

The Agro^®^ Hydrogel morphology was recorded using scanning electron microscopy (Quanta 3D FEG, FEI, Hillsboro, OR, USA) to characterize the adsorbent thoroughly before and after the adsorption. 

The thermal analysis was performed using Q50 TGA (TA Instruments, New Castle, DE, USA) to determine the characteristic temperature from the thermogravimetric curves. 

ASAP 2040 (Micromeritics, Norcross, GA, USA) analyzer was used for nitrogen adsorption/desorption isotherms determination. 

The pH and pH_ZPC_ values were measured using the analytical and measuring set (Metrohm, Zofingen, Switzerland) according to the detail described in [26]. The pH of 0.01 M NaCl solution of Agro^®^ Hydrogel was adjusted between 2 and 12 by adding 0.1 M NaOH and 0.1 M HCl. 0.2 g of Agro^®^ Hydrogel was added, and after 24 h, the final pH value was measured. The procedure was repeated three times with the reproducibility of 5% to obtain the mean value. 

### 2.3. Adsorption and Kinetic Equilibrium

Incorporation experiments were performed by the batch study. The adsorption efficiency of metals with the complexing agents (IDHA, EDDS, or GLDA) was determined by taking into account various parameters such as contact time (1–240 min), mass (0.05–0.25 g), concentration (1 × 10^−3^ M), pH (2–12), temperature (293–333 K). At first, 0.1 g of Agro^®^ Hydrogel and 50 mL of Cu(II), Zn(II), Mn(II) and Fe(III) complexes with IDHA, EDDS and GLDA (in the system M(II)/(III): IDHA, EDDS or GLDA = 1:1) were shaken at 180 rpm at 293 K in a laboratory shaker of type 357 (Elpin Plus, Lubawa, Poland). The concentration in the filtered solutions was determined using an inductively coupled plasma optical emission spectrometer (ICP-OES 72O ES, Varian Inc.), and the amount q_t_ (mg/g) of adsorbate adsorbed by the unit mass of Agro^®^ Hydrogel was calculated as:

q_t_ = V(c_0_ − c_e_)/m (1)

where c_0_ is the initial concentration of adsorbate in the aqueous phase (mg/L or M); c_e_ is the equilibrium concentration (mg/L); V is the volume of the solution (L), and m is the mass of Agro^®^ Hydrogel (g). 

### 2.4. Instruments

Spectrometer ICP-OES of 720 ES (Varian, Melbourne, Australia) with a Conikal^®^ nebulizer and a cyclonic spray chamber was used to measure the concentration of Cu(II), Zn(II), Mn(II), and Fe(III) complexes with IDHA, EDDS, and GLDA in the filtrate. The following were the instrumental settings and operating circumstances used for the ICP-OES techniques determination of the examined elements: power 1200 W, plasma argon gas flow rate 15 mL/min, auxiliary argon gas flow rate 2.25 L/min, nebulizer argon gas flow rate 0.2 L/min, pomp rate 12 rpm as well as analytical wavelength for Cu 327.395 nm, for Zn 213.857 nm, for Mn 257.610 nm and for Fe 259.940 nm. 

Scanning electron microscopy Quanta 3D FEG (FEI, USA) was used to characterize the adsorbent thoroughly before and after the adsorption. The FEI Quanta 3D supports research to fabricate samples for transmission electron microscopy and scanning transmission electron microscopy (TEM/STEM) analyses and 3D-atom probe tomography. Its focused ion beam (FIB) scanning electron microscope (SEM) is equipped with energy-dispersive X-ray spectroscopy (EDS) and electron backscatter diffraction, or EBSD. At 30 keV, high-resolution surface imaging is feasible down to 1 nm, and it can work in high, low, and environmental vacuum modes.

FTIR Carry 630 spectrometer (Agilent Technologies, Santa Clara, CA, USA) was used to identify the characteristic functional groups present on the hydrogel surface. The FTIR-ATR technique was applied to measure the characteristic band at the wavenumber ranging from 4000 to 550 cm^−1^ with a resolution of 8 cm^−1^.

All data were processed using Microsoft Excel 2010 Microsoft Corp. (Redmond, WA, USA) and Origin Pro8 (OriginLab Corp., Northampton, MA, USA).

## 3. Results and Discussion

### 3.1. Agro^®^ Hydrogel Characterization

Water and fertilizers have a positive impact on agricultural production. With the controlled release of fertilizers, the main method is to cover a conventional soluble fertilizer with a protective coating that is insoluble in water, semi-permeable, or impermeable with porous materials. Thus, slow-release fertilizers have many advantages over conventional ones [37]. As fertilizer coatings for the production of polymer-coated fertilizers (PCFs), polyacrylamide, polyacrylic acid, polymethacrylic, polyvinyl alcohol, and polymers based on polysaccharides such as chitosan, pectine and carbon-methylcellulose are used [38,39]. Fertilizers coated with polysulfone, polyvinyl chloride, and polystyrene are also known. However, in this case, after the release of fertilizers, the remaining coating materials are difficult to degrade [39]. Therefore, of significant importance are the materials that release nutrients, have incorporated into matrix nutrients, and possess a biodegradable coat. The soil solution caused the hydrogel coating to swell, which aided in the interactions of polymers with soil microorganisms and led to hydrogel degradation. In this field, the main role played by hydrogels is mainly to release water and nutrients into the soil without causing environmental pollution [40]. Chemicals trapped in the polymer network or other structures are not washed out immediately by water, but they are gradually released into the soil and absorbed directly by plants. Therefore, environmentally safe and biodegradable materials are desired and used. What is more, diffusion through the semipermeable polymer surface is controlled by varying the composition and thickness of the coatings. These affect the fertilizer loss rates and supply micronutrients sustainably. According to micronutrient release research, the double-coated fertilizer released around 81.4% of N and 41.2% of Zn after 30 days, indicating that the coated fertilizer can limit nutrient release rates effectively [39]. Moreover, the cross-linker concentration (producing cross-linked points in polymeric chains) and increasing the extent of cross-linking of the polymer network are also important [41]. Soil amendments also have a positive impact on agricultural production. They encompass a wide range of organic and inorganic substances tailored to improve soil structure, fertility, drainage, and nutrient content [3]. These materials are strategically introduced to improve soil capacity to support healthier plant growth, increase crop yields, and foster a more sustainable ecosystem. Soil amendments can vary in their composition and function, serving purposes such as enhancing nutrient, improving soil structure, aid in soil aeration, enhancing moisture retention and ensuring better root growth and nutrient uptake. Adjusting pH levels, altering drainage and promoting microbial activity as well as reducing soil erosion are also very important. Therefore, in the paper, the commercially available Agro^®^ Hydrogel was used. Hydrogels based on acrylate and acrylamide, such as Agro^®^ Hydrogel, can be synthesized by copolymerization of the mixture of acrylate (AA) and acrylamide (AM) or base hydrolysis of acryloamide to obtain [P(AA-co-AM)]. In the linear anionic copolymer of acrylate and nonionic acrylamide monomers, the ratio of acrylic to acrylamide groups on the polymer chain can be varied, and in the presented studies, this was not verified (the data of the manufacturer are not available) either. The structure and molecular weight of acryloamide are almost the same as those of acrylic acid. It is also water-soluble [42]. Such polymers are pH-responsive because they have functional groups: non-ionic basic (such as amine) or acidic (such as ionic carboxylic) that release or obtain protons in response to the changes in the pH values. In an acidic medium, the -COO^−^ groups can react with H^+^, and in an alkaline medium, -COOH can react with OH^−^ of the soil solution. The results indicate that the hydrogel coating can buffer soil acidity or alkalinity and develop an optimal pH for plants. Moreover, the presence of ionic groups in polymer chains results in increasing swelling. Another issue is the cross-linking. It is well-known that the higher the cross-linker concentration (more cross-linked points in the polymer net), the less swelling. It was proved that the hydrogels hydrolyzed with the NaOH solution are characterized by larger swelling capacities at the higher initial AM/AA ratios [41]. Therefore, they are still investigated for possible uses in slow-release fertilizer systems.

**Figure 1:**
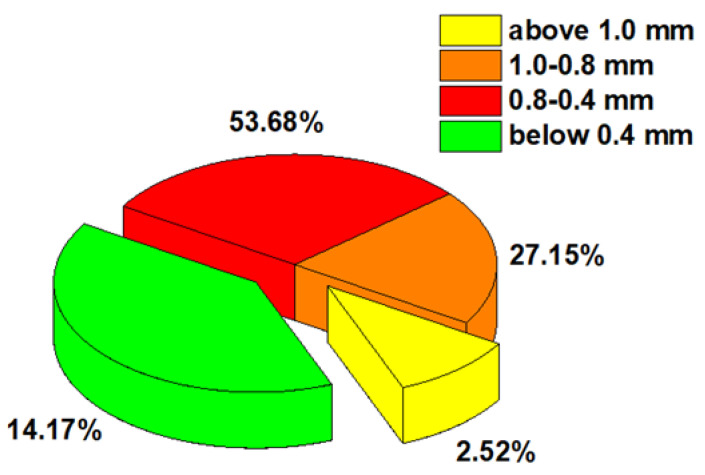
Grain composition of Agro^®^ Hydrogel.

The physicochemical properties, as well as the macro- and microscans of Agro® Hydrogel, are presented in Table S1. The images obtained from the scanning electron microscope allow the analysis of the size and shape of individual grains and agglomerates. As for Agro^®^ Hydrogel, the grain composition diagram indicates that the values of bead size obtained vary from those declared by manufacturers (Figure 1). 

It is better to understand the capability of Agro® Hydrogel to hold or release water and different salts, taking into account their swelling characteristics, which are a crucial aspect. A swelling ratio is defined as the proportion of the hydrogel weight that increases as a result of water/salt absorption because the structure of the hydrogel can expand and shrink reversibly. 

**Figure 2:**
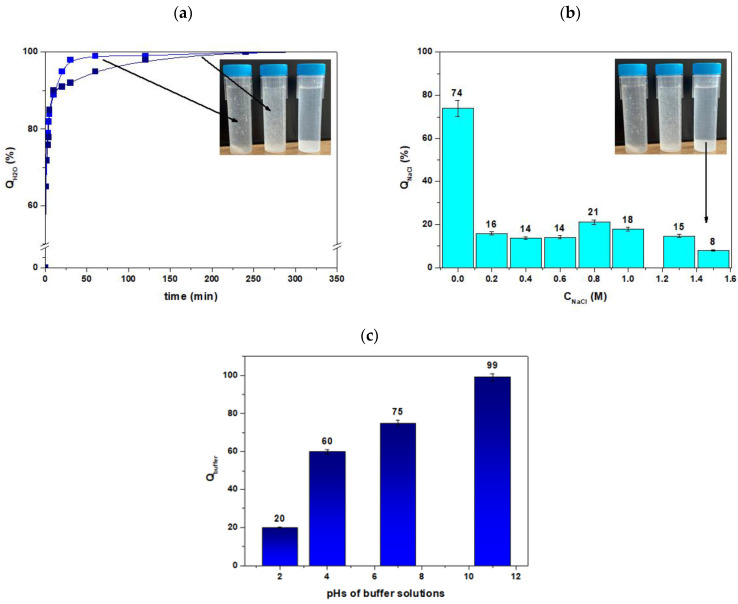
Comparison of the moisture retention capability of Agro^®^ Hydrogel for (**a**) distilled (blue) and tap water (navy) and (**b**) NaCl and (**c**) buffers.

As for the main properties of Agro^®^ Hydrogel, it was found that the G% and M% values were equal to 98% and 80% (after 24 h). It was also found that Q_H2O_,% and Q_NaCl_,% (Supplementary Materials) were equal to 99% and 74%, respectively. Moreover, analogous results were obtained for the tap water. The effect of pH on the swelling was also studied in buffer solutions (pH 2, 4.01, 7, and 11). The procedures were the same as those described above. The obtained results are presented in Figure 2. It should be noted that Q_NaCl_, % decreases as the concentration of NaCl increases.

The performance of the Agro^®^ Hydrogel in the NaCl solution (generally in the salt solution) was significantly reduced compared to the values measured in the tap water and deionized water. This well-known phenomenon, which is usually observed in ionic hydrogel agglomeration, is often attributed to the additional cation charge screening effect, which causes an incomplete anion-anion electrostatic repulsion, resulting in a decrease in the osmotic pressure difference (ionic pressure) between the hydrogel network and the external solution. Changing the NaCl concentration to more than 1.5 M has no appreciable influence on absorbency of the hydrogel. The analogous experiments were performed also for the NaOH solution (not presented). It was found that Q_NaOH_ increases with the increasing NaOH, probably due to the increase of the hydrophilic functional groups. 

For the description of the pH effect, it was found that the two sharp swelling capacity changes can be attributed to great repulsion of –NH_3_^+^ groups in acidic media and –COO^−^ groups in basic media. As for the pH effect, it was found that the two sharp swelling capacity changes can be attributed to the great repulsion of –NH_3_^+^ groups in acidic media and –COO^−^ groups in basic media. As follows from the literature data, the pKa of the acrylate acid is equal to 4.25 [43]. At this value, both the base and acid groups are non-ionized. It is commonly known that the intrinsic dissociation constant of the carboxylic acid depends on the nature of its chemical neighborhood, and such an effect is probably particularly pronounced when the function is incorporated along a polymeric chain. According to [44], when the acrylic unit is surrounded by acrylamide groups, the value of pK_a_ of acrylic acid is around 4 (polyacrylic acid at high ionization degree is equal to 6.4), and it rises if one or two adjacent groups are acrylic units. At higher pHs (7 and 11), the carboxylic acid groups become ionized, and increasing swelling is observed. Protonation of amine and carboxylic groups takes place at pH < 4.7. These groups increase the charge density of the polymer and cause an increase in the osmotic pressure within the gel particles due to the electrostatic repulsion. The osmotic pressure difference between the inner solution and the outer solution of the network is balanced by the influx of the gel.

**Figure 3:**
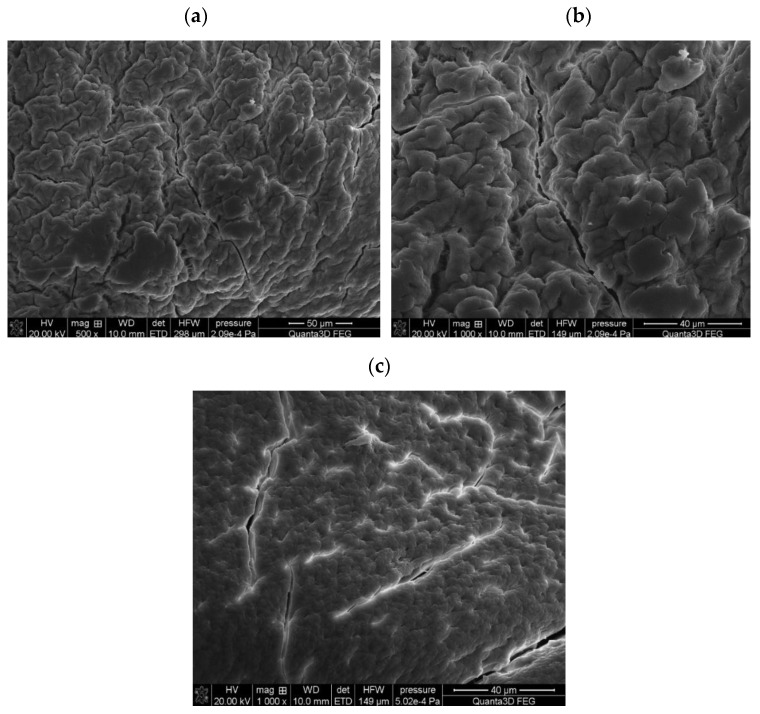
SEM images of Agro^®^ Hydrogel at different modifications (500×, 1000×) before (**a**,**b**) as well as after (**c**) the process of Cu-IDHA complexes incorporation.

Scanning electron microscopy (SEM) was used to examine the Agro^®^ Hydrogel structural topography. Figure 3 shows the gel surface at lesser and higher magnification (500× and 1000×). It was evident from these SEM images that the hydrogel had a porous structure. The gel was able to absorb large amounts of water/salt owing to its porous nature. By means of intermolecular chemical forces, the added solvent or molecules could interact with the hydrogel network.

**Figure 4:**
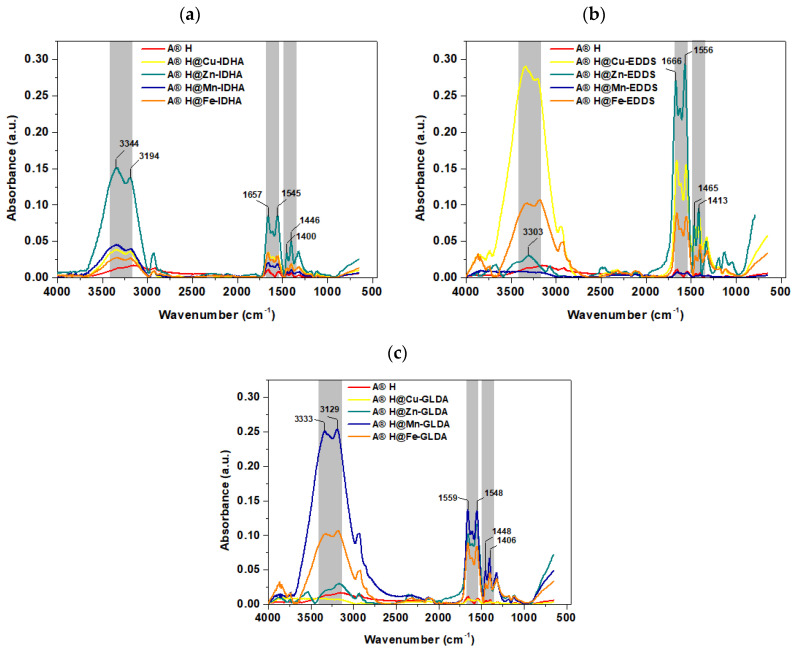
FTIR-ATR spectra of Agro^®^ Hydrogel before and after the adsorption of Cu(II), Zn(II), Mn(II), and Fe(III) complexes with (**a**) IDHA, (**b**) EDDS, and (**c**) GLDA.

Before and after the incorporation process of Cu(II), Zn(II), Mn(II), and Fe(III) complexes with IDHA, EDDS, and GLDA (Figure 4), FTIR-ATR spectra and characteristic Agro^®^ Hydrogel bands were recorded. 

The bands at 3344–3333 and 2928 cm^−1^ were due to the –OH and C–H stretching vibrations of both acrylate and acrylamide matrices. It is evident that for the studied hydrogel, two N–H stretching bands appear at 3344 and 3194 cm^−1^, respectively. After the sorption of Cu-IDHA, Cu-EDDS, and Cu-GLDA complexes on Agro^®^ Hydrogel C = O stretching vibrations of COOH at 1727 cm^−1^ disappear, and the presence of bands assigned to symmetric stretching of COO^–^ groups (1413–1400 cm^−1^), asymmetric stretching of carboxylate groups (1465–1446 cm^−1^) are visible. Moreover, the band at 1658 cm^−1^ for the Agro^®^ Hydrogel spectrum is not much stronger than that at 1657, 1666, and 1559 cm^−1^ for Agro^®^ Hydrogel@Cu-IDHA, Agro^®^ Hydrogel@Cu-EDDS or Agro^®^ Hydrogel@Cu-EDDS spectrum, which is mainly attributed to the stretching vibrations of C = O bonds [45]. The band at 1464–1446 cm^−1^ is the stretching band of C–N in the CONH_2_ group, and 1112 cm^−1^ is another band related to the amide group. The stretching vibrations of the -CH_2_ group can also be assigned at 1465 cm^−1^. The part of the characteristic peak at 615 cm^−1^ originated from the –OH out of plane related to the carboxylic groups of acrylate branches on the polymeric chains is also evident.

**Figure 5:**
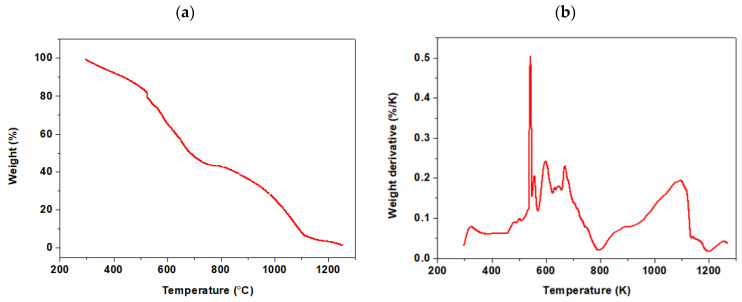
Comparison of (**a**) TGA and (**b**) DTG curves of Agro^®^ Hydrogel.

The thermogravimetric analysis (TGA) of the hydrogel showed that over the entire heating temperature range (up to 1200 K), four basic areas characterizing the change in the sample mass can be distinguished. TGA and DTG curves are presented in Figure 5.

The first loss of mass is associated with the evaporation of water and ranges from 5% in the first stage, starting from room temperature to approx. 550 K. Another loss of mass is associated with the slow, gradual decomposition of the polymer to approx. 650 K. In the temperature range of 500–800 K, decomposition of the side chains can occur, and in the range of 800–1200 K, complete decomposition of the main chain of the hydrogel can occur, i.e., combustion of the organic substance up to about 60% of the sample weight. For Agro^®^ Hydrogel, which has a polyacrylamide group, there was observed the presence of sharp peaks at about 475 K and 540 K can be related to the thermal decomposition of the amide group and side carboxyl groups of the polymer chain, respectively.

### 3.2. Effect of Adsorbent Mass 

**Figure 6:**
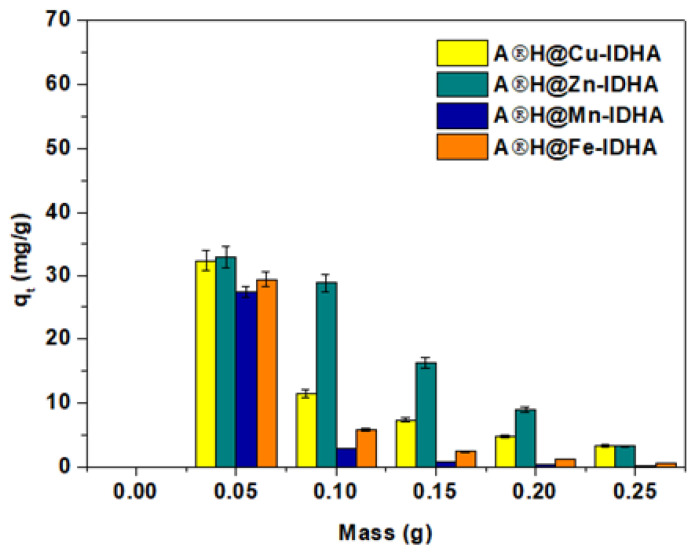
Effect of Agro^®^ Hydrogel mass on adsorption of Cu(II), Zn(II), Mn(II), and Fe(III) complexes with IDHA (C_0_ 1 × 10^−3^ M, sorbent mass 0.05–0.25 g, phase contact time 240 min, shaking 180 rpm).

The adsorption capacities with a different mass of Agro^®^ Hydrogel (0.05, 0.1, 1.5, 0.2, and 0.25 g) and other constant parameters were studied to describe the effect of adsorbent mass and to optimize the minimum dosage required for the effective adsorption process of Cu(II), Zn(II), Mn(II) and Fe(III) complexes with IDHA, EDDS or GLDA. As an example, the effect of different adsorbent masses on the IDHA complexes sorbed on Agro^®^ Hydrogel is shown in Figure 6. The adsorption capacities decreased significantly with the increasing adsorbent dose. 

The decreased adsorption capacity with the increase of Agro^®^ Hydrogel dosage can be assigned by the agglomeration of its particles and the abundant active sites available for adsorbate sorption when the adsorbent concentration is increased while keeping the concentration of the adsorbate constant.

### 3.3. Effect of Initial pH

**Figure 7:**
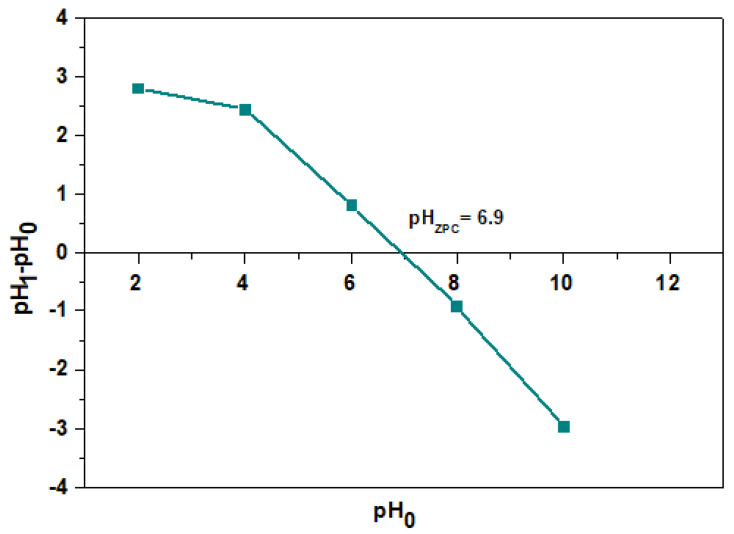
pH_ZPC_ of Agro^®^ Hydrogel.

As follows from Figure 7, pH_ZPC_ was determined to be equal to 6.9. At pH > pH_ZPC_, the surface charge is negative; however, at pH < pH_zpc_, it is positive. It was found that the adsorption process proceeds at a pH value smaller than that of pH_ZPC_, which indicates that the charge on the beads’ surfaces of the obtained sorbents was positive.

**Figure 8:**
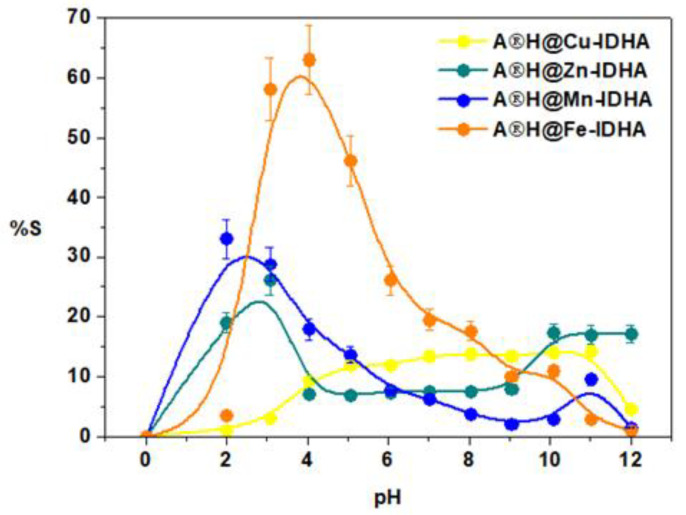
Percentage removal of Cu(II), Zn(II), Mn(II), and Fe(III) complexes with IDHA at various pH values with Agro^®^ Hydrogel (C_0_ 1 × 10^−3^ M, sorbent mass 0.1 g, phase contact time 240 min, shaking 180 rpm).

The percentage removal of Cu(II), Zn(II), Mn(II), and Fe(III) complexes with IDHA at various pH values with Agro^®^ Hydrogel is plotted in Figure 8. For the Cu(II) and Zn(II) complexes, the adsorption percentage (S%) increases slightly at the pH values ranging from 4 to 10. However, the effect for the Mn(II) and Fe(III) complexes is opposite and noticeable, especially for Fe(III) complexes. This indicates a different mechanism of adsorption or adsorption of different types of complexes, such as [M(idha)]^2-^ for Mn(II) or [M(idha)]^−^ for Fe(III). It was also demonstrated that the adsorption efficiency of Zn(II) complexes with IDHA increased as the pH increased from 10 to 12. Adsorption was generally more favorable at the pH values ranging from 4 to 10 but decreased dramatically at the pH value 2, especially for Fe(III). In the case of hydrogels the electrostatic interactions, hydrogen bonding, π-π interactions, ion exchange, surface complexation and coordination/chelation should be noted. It is worth mentioning that several mechanisms exist in the adsorption process generally, depending on both the composition of hydrogels and the set adsorptive condition (pH, temperature, concentration, etc.).

With the stability constants presented in Table 1, IDHA, EDDS, and GLDA form the 1:1 complexes with M(II) and M(III). 

According to these data, the highest values have been established for EDDS for the individual metal complexes. The highest value was found for Fe(III). The stable constant values of metal complexes with IDHA are comparable with those of GLDA [46]. The obtained complexes have an octahedral structure, which is sometimes completed by the water molecules [39]. For example, for Cu(II) ions, there are known the following complexes: [Cu(Hidha)]^−^, [Cu(idha)]^2−^ and [Cu(OH)(idha)]^3−^. It should be emphasized that as a new generation of complexing agents, they are characterized by a higher biodegradability compared with conventional agents, such as, for example, EDTA. They are non-toxic, especially in the aquatic environments. From the values of the stability constants presented in Table 1, it can be concluded that decomposition of their complexes in the adsorbent phase is possible but only at small pH values.

### 3.4. Kinetic Studies

**Figure 9:**
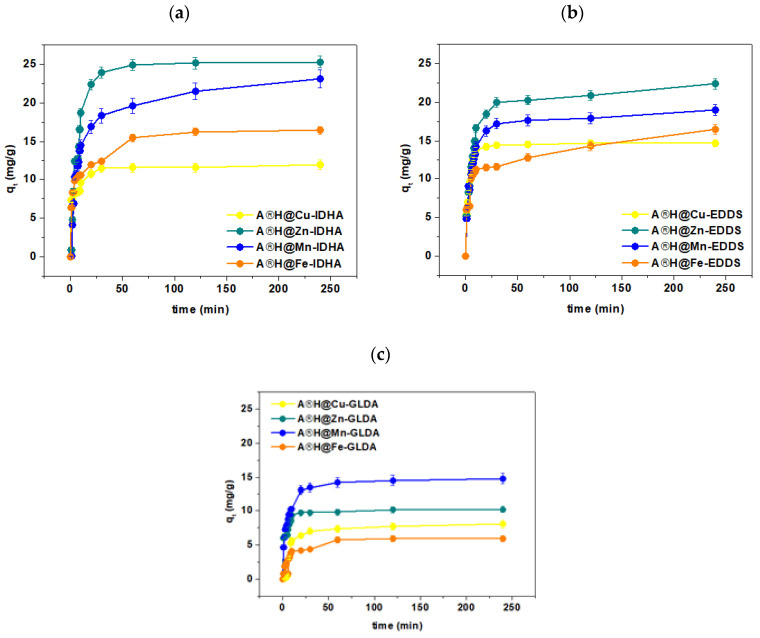
Effect of the phase contact time on the sorption capacity for the Cu(II), Zn(II), Mn(II), and Fe(III) complexes (C0 1 × 10^−3^ M, sorbent mass 0.1 g, phase contact time 240 min, shaking 180 rpm).

The kinetic results showed that equilibrium was attained after about 60 min (Figure 9). Based on the PFO and PSO models (Supplementary Materials), the kinetic parameters were determined, and they are listed in Table 2 to determine the mechanism of Cu(II), Zn(II), Mn(II), and Fe(III) ion adsorption in the presence of IDHA, EDDS, and GLDA.

The PSO model was found to be suitable for describing the adsorption process because of (i) the linear relationship t/qt = f(t), (ii) the values of the coefficients of determination (R^2^) are close to one, and (iii) there is good agreement with the experimental data (q_e2_ values are almost equal to q_e_,_exp_). The maximum adsorption capacities with respect to the tested complexes in the case of Agro^®^ Hydrogel were as follows: 11.99 mg/g for Cu(II)-IDHA, 22.14 mg/g for Zn(II)-IDHA, 14.39 mg/g for Mn(II)-IDHA and 16.24 mg/g for Fe(III)-IDHA. The amounts of sorbed complexes after 240 min calculated based on the PSO model on the Agro^®^ Hydrogel are Cu(II)-IDHA 12.12 mg/g, Zn(II)-IDHA 25.91 mg/g, Mn(II)-IDHA 23.61 mg/g, Fe(III)-IDHA 16.82 mg/g. For GLDA complexes they are as follows: Cu(II)-GLDA 8.74 mg/g, Zn(II)-GLDA 10.31 mg/g, Mn(II)-GLDA 15.07 mg/g, Fe(III)-GLDA 6.23 mg/g and for EDDS complexes: Cu(II)-EDDS 14.74 mg/g, Zn(II)-EDDS 22.64 mg/g, Mn(II)-EDDS 15.96 mg/g, Fe(III)-EDDS 16.39 mg/g. There were determined for Agro^®^ Hydrogel for the following affinity series: Cu(II): IDHA > EDDS > GLDA; Zn(II): IDHA > EDDS > GLDA; Mn(II): EDDS > IDHA > GLDA; and Fe(III): IDHA > EDDS > GLDA. 

**Figure 10:**
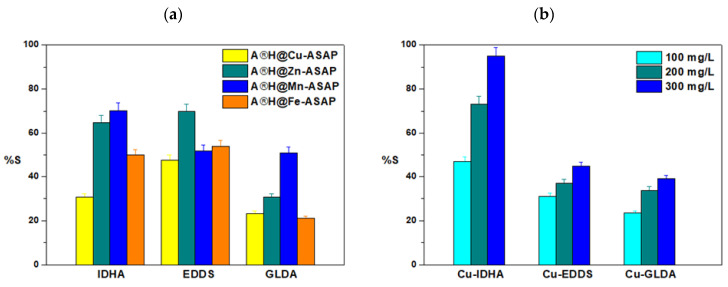
Comparison of the adsorption percentage (S%) of (**a**) Cu(II), Fe(III), Mn(II), and Zn(II) complexes with EDDS, IDHA, and GLDA on Agro^®^ Hydrogel depending on the phase contact time, (**b**) Cu(II) complexes with EDDS, IDHA, and GLDA on Agro^®^ Hydrogel depending on the concentration of the solution (C0 1 × 10^−3^ M, sorbent mass 0.1 g, phase contact time 240 min, shaking 180 rpm).

The reaction rate coefficient, k_2_, ranges from 0.006 to 0.069 (g/mg min). The studies show that the concentration affects the adsorption percentage (S%) of Cu(II), Zn(II), Mn(II), and Fe(III) complexes with IDHA, EDDS, and GLDA on Agro^®^ Hydrogel (Figure 10a). It was found that the efficiency of the adsorption process (S%) increases with the increasing concentration of the starting solution from 100 mg/L to 300 mg/L (Figure 10b). 

The experimental kinetics of Cu(II), Zn(II), Mn(II), and Fe(III) complexes with IDHA, EDDS, and GLDA on Agro^®^ Hydrogel shows a sharp increase in the first few minutes, followed by a slower rate in the following stage. The initial part depends on the faster mass transfer through the boundary layer and/or adsorption on the solid surface, followed by slow diffusion inside the particles. The Morris and Weber model predicts the k_i_ values ranging from 0.26–6.14 mg/g min1/2 for the Cu(II), Zn(II), Mn(II), and Fe(III) complexes with IDHA, EDDS, and GLDA adsorption on Agro^®^ Hydrogel.

### 3.5. Temperature Effect, Adsorption and Thermodynamic Calculations

**Figure 11:**
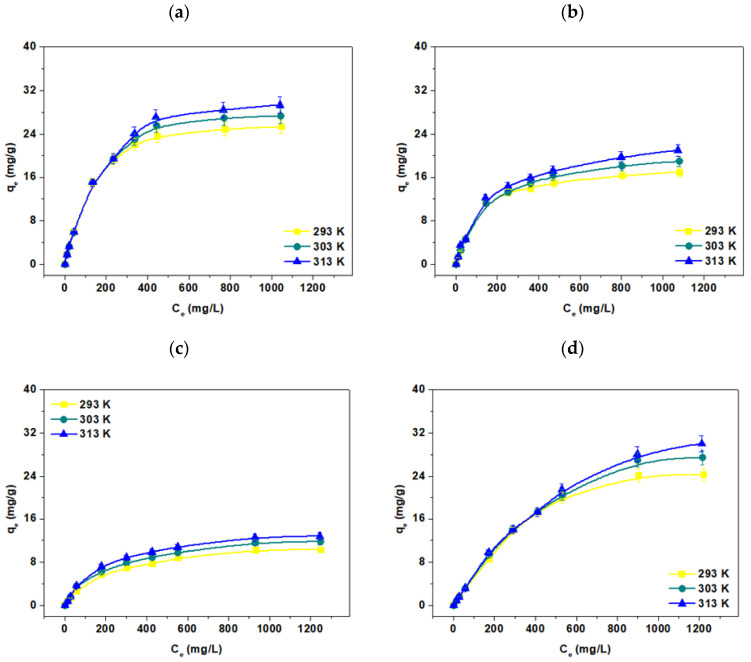
Effect of temperature on (**a**) the Cu(II), (**b**) Zn(II), (**c**), Mn(II), (**d**) Fe(III) complexes with IDHA on Agro^®^ Hydrogel (C_0_ 0.25 × 10^−3^–2 × 10^−2^ M, sorbent mass 0.1 g, phase contact time 120 min, shaking 180 rpm).

The static method was used to investigate the effect of temperature in the range of 293–313 K on the sorption process of Cu(II), Zn(II), Mn(II), and Fe(III) ions in the presence of IDHA on Agro^®^ Hydrogel. Temperature is another parameter that has a large impact on the efficiency of the metal ion sorption process. The obtained data are presented in Figure 11. 

The tests were carried out for the initial concentrations in the range of 0.25 × 10^−3^–2 × 10^−2^ M, the phase contact time 120 min, the shaking speed 180 rpm, and the amplitude 7. From the linear relationships C_e_/q_e_ vs. C_e_ (Langmuir isotherm), logq_e_ vs. logC_e_ (Freundlich isotherm), q_e_ vs. lnC_e_ (Dubinin-Radushevich isotherm), lnq_e_ vs. ε^2^ (Temkin isotherm) there were calculated the following constants: Langmuir q_0_ and K_L_, Freundlich KF and 1/n, Temkin K_T_ and b_T_ and Dubinin-Raduszkiewicz Xm and β. They are presented in Table 3. 

The results prove that the Langmuir isotherm model describes the sorption of Cu(II), Zn(II), Mn(II), and Fe(III) complexes with IDHA on Agro^®^ Hydrogel precisely. This is evidenced by the high coefficients of determination (R^2^). Similar results were presented in [47,48,49,50,51,52,53,54]. A good fit of the experimental data to the linear form of the Langmuir isotherm proves that a monolayer is formed during the sorption of Cu(II), Zn(II), Mn(II), and Fe(III) complexes with IDHA and EDDS. This model is based on the assumption that the solid adsorbents have particularly active sites on their surfaces, called active centers, where the adsorption process takes place. Each center can adsorb only one molecule, i.e., the adsorbent is covered with a monomolecular layer. Moreover, the molecules adsorbed on the adsorption centers do not interact with each other, and the process itself is a dynamic equilibrium between the adsorption and desorption. This equation describes the chemical adsorption particularly well. The Langmuir adsorption isotherm is a fundamental adsorption equation that can be considered the starting equation for many more complex equations. 

In turn, the Freundlich isotherm is described by the empirical equation in which the mass of the adsorbed substance and the fractional value of the exponent n are presented. This parameter provides information about the sorption process intensity, and its inverse determines the degree of diversity of active centers on the sorbent surface in the range 0 < 1/n < 1. When 1/n is close to zero, the sorbent shows a significant homogeneity of the surface. Moreover, when the 1/n values are between 0 and 1, a non-linear relationship between the solution concentration and the sorption is observed. If the values of 1/n are equal to 1, the sorption process can be described by the linear equation [50]. For the tested systems, these values are in the following ranges: 0.34–0.37 for the Cu(II), Zn(II), Mn(II), and Fe(III) complexes with IDHA on the Agro^®^ Hydrogel. It was also proved that for Agro^®^ Hydrogel, the value of the K_F_ parameter increases with the increasing temperature. However, for all the studied systems, the fit of the experimental data for the Freundlich isotherm model is worse than for the Langmuir isotherm one, which is confirmed by the smaller values of the determination coefficients. 

The two-parameter Temkin isotherm model is used to describe the adsorption of the monolayer onto the heterogeneous surface. This isotherm corresponds to the continuous, infinite distribution of adsorption sites. It is assumed that the heat of adsorption of all molecules in the layer decreases linearly and that adsorption is characterized by the even distribution of bonding energies. In the sorption studies of Cu(II), Zn(II), Mn(II), and Fe(III) complexes with IDHA on the Agro^®^ Hydrogel, small values of the determination coefficients were obtained. In addition, the calculated parameters show a much worse degree of fit to the experimental data compared to the Langmuir isotherm model. Therefore, the Temkin isotherm model is not suitable for describing the sorption process of metal complexes on the tested hydrogels. Small heat values of the sorption process cause energy to be supplied to the system (endothermic process). The b_T_ values are smaller than 10.00 J/mol and also indicate low efficiency of the sorption process. 

For the value of sorption energy (E) in the range of 8–16 kJ/mol, according to the Dubinin-Radushevich model, the ion exchange mechanism dominates. When E < 8 kJ/ mol, the process is characterized by the physical sorption, which is caused by the van der Waals interactions [51]. Based on the results obtained at 333 K, it can be concluded that the ion exchange takes place in the case of most of the tested complexes on Agro^®^ Hydrogel. The experimental data fit coefficients to the linear lnqe vs. ε^2^ are for the Cu(II)-IDHA, Zn(II)-IDHA, Mn(II)-IDHA, and Fe(III)-IDHA complexes on Agro^®^ Hydrogel: 0.9962, 0.9467, 0.9697, 0.9633, respectively. The thermodynamic characterization of Cu(II), Zn(II), Mn(II), and Fe(III) complexes with IDHA was performed to better understand the processes occurring during their sorption. The basic thermodynamic parameters are presented in Table 4. For all tested systems, the relationship between lnK_c_ vs. 1/T was the basis on which the entropy change (ΔS^o^) and the enthalpy change (ΔH^o^) were calculated.

As follows from the analyses, the ΔG^o^ values of all tested systems are negative and do not exceed −20 kJ/ mol. The negative ΔG^o^ values higher than −20 kJ/ mol mean that the process is dominated by physical adsorption. The negative free energy results obtained mean that the sorption of Cu(II), Zn(II), Mn(II), and Fe(III) complexes with IDHA on Agro^®^ Hydrogel is a spontaneous process. In addition, for the sorption process of all investigated complexes, there was an increase in ΔG^o^ with the increasing temperature from 293 to 333 K. During the sorption studies of Cu(II) on the hydrogel with the polyacrylamide matrix carried out by Chen et al. [50], the obtained ΔG^o^ values were also smaller than −20 kJ/mol. Moreover, the obtained ΔH^o^ values for the sorption of Cu(II), Zn(II), Mn(II), and Fe(III) complexes with IDHA are in the range of 2.62–5.88 kJ/mol, also indicating the physical adsorption. Small enthalpy values were obtained for most of the studied systems, which also indicates that, in these cases, the interactions between the above-mentioned complexes and hydrogels are weak. The sorption process of Cu(II), Zn(II), Mn(II), and Fe(III) complexes with IDHA on Agro^®^ Hydrogel hydrogel is endothermic, which is confirmed by the positive ΔH^o^ values. In turn, the negative values of ΔS^o^ obtained for all tested systems indicate a decrease in the state of disorder in the solid-liquid system. Similar research results were obtained in [55,56,57,58].

### 3.6. Desorption Studies

Agro^®^ Hydrogel was collected, dried, and transferred to several 100 mL conical flasks with a 50 mL desorbing agent and physically shaken using the laboratory shaker following the equilibrium research with the initial concentration of 1200 mg/L Mn(II)-EDDS. In one series, there are five desorbing agents, distilled water, 0.1 M HNO_3_, 0.1 M HCl, 1 M HNO_3_, and 0.1 M HCl. For 24 h, the samples were stirred at 180 rpm. Measurements were made of the Mn(II) ion concentrations in the eluate (Table 5). The following equation was used to calculate the desorption of Mn(II) from hydrogels: D% = the amount of desorbed M(II)/the amount of adsorbed Mn(II) × 100. It was found that the best desorption conditions were found using 1 M HCl and 1 M HNO_3_.

## 4. Conclusions

This research presents the latest reports on the influence of chemical conditions on superabsorbent kinetic and adsorption behavior towards metal ions in the presence of the chelating agents of a new generation, namely, IDHA, EDDS, and GLDA. To sum up the obtined results, the sorption efficiency of Cu(II), Zn(II), Mn(II) and Fe(III) complexes with IDHA, EDDS and GLDA on Agro^®^ Hydrogel is affected by pH, contact phase time, temperature, concentration and complexing agent type. The efficiency of the process increases with the increasing phase of contact time. The kinetic results showed that equilibrium was attained after about 60 min. The sorption capacity sequence was as follows: Cu(II): IDHA > EDDS > GLDA; Zn(II): IDHA > EDDS > GLDA; Mn(II): EDDS > IDHA > GLDA and Fe(III): IDHA > EDDS > GLDA for Agro^®^ Hydrogel. The process is also pH-dependent. Both complexes of Mn(II) and Zn(II) exhibit a high affinity for Agro^®^ Hydrogel. These results were confirmed by the FTIR-ATR studies and SEM analysis. The PSO mechanism reaction is used to adsorb the Cu(II), Zn(II), Mn(II), and Fe(III) complexes with IDHA, EDDS, and GLDA onto Agro^®^ Hydrogel. The adsorption mechanism can be described by the Langmiur equation. The promising results obtained can be the basis for the development of innovative ways to fertilize plants and thus increase productivity.

## Figures and Tables

**Table 1:** The comparison of the stable constants (log K) of Cu(II), Fe(III), Zn(II), and Mn(II) complexes with IDHA, EDDS, and GLDA in the M(II/III)-L = 1:1 system as well as other metal complexes.

** 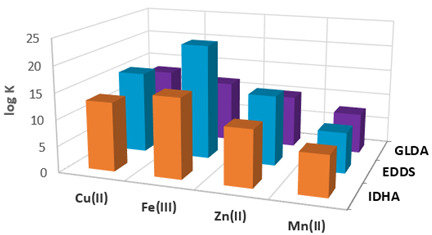 **			
M(II)/(III)	IDHA	EDDS	GLDA
Cr(III)	9.6	n.a.	n.a.
Hg(II)	14.9	n.a.	14.3
Mg(II)	6.9	6.0	6.1
Ni(II)	12.2	16.7	10.9
Pb(II)	11.0	12.7	10.5

where n.a.—not available.

**Table 2:** The kinetic parameters for the sorption of Cu(II), Zn(II), Mn(II), and Fe(III) complexes with IDHA, EDDS, and GLDA on Agro^®^ Hydrogel.

Complexing Agent	M(II)/(III)	Pseudo-First Order			Pseudo-Second Order			Intraparticle Diffusion	
		q_e1_ mg/g	k_1_ 1/min	R^2^	q_e2_ mg/g	k_2_ g/mg min	R^2^	k_i_ mg/g min	R^2^
IDHA	Cu(II)	3.62	0.025	0.7009	12.12	0.026	0.9998	3.14	0.7666
	Zn(II)	14.02	0.045	0.8879	25.91	0.008	0.9994	6.14	0.8711
	Mn(II)	13.53	0.020	0.8437	23.61	0.005	0.9991	0.90	0.8567
	Fe(III)	8.16	0.031	0.9836	16.82	0.011	0.9991	4.14	0.9284
EDDS	Cu(II)	4.48	0.074	0.9385	14.74	0.069	0.9999	4.71	0.9962
	Zn(II)	10.51	0.020	0.7056	22.64	0.008	0.9993	4.90	0.9869
	Mn(II)	4.85	0.016	0.9523	15.96	0.021	0.9989	4.62	0.9813
	Fe(III)	7.54	0.011	0.7214	16.39	0.009	0.9935	0.44	0.9893
GLDA	Cu(II)	5.80	0.029	0.7944	8.74	0.006	0.9318	0.24	0.8519
	Zn(II)	2.85	0.034	0.8346	10.31	0.047	0.9999	2.57	0.7728
	Mn(II)	6.61	0.031	0.8646	15.07	0.016	0.9999	3.52	0.9704
	Fe(III)	5.01	0.060	0.9751	6.23	0.015	0.9773	0.27	0.8876

**Table 3:** Adsorption isotherm parameters for the adsorption of the Cu(II), Fe(III), Mn(II), and Zn(II) complexes with IDHA on Agro^®^ Hydrogel.

Isotherm Models	Parameters	Cu(II)	Zn(II)	Mn(II)	Fe(III)
Langmuir	q_0_	35.33	56.01	60.77	43.69
	K_L_	0.012	0.017	0.019	0.013
	R^2^	0.9957	0.9988	0.9970	0.9942
Freundlich	K_F_	3.42	5.43	6.59	3.65
	1/n	0.34	0.36	0.34	0.37
	R^2^	0.9810	0.8992	0.9336	0.9277
Temkin	K_T_	0.256	0.346	0.483	0.282
	b_T_	5.864	8.693	9.667	7.282
	R^2^	0.9895	0.9871	0.9691	0.9942
Dubinin-Raduszkiewicz	q_m_	53.48	100.87	104.83	85.70
	β	0.0040	0.0046	0.0043	0.0048
	E_a_	10.71	10.42	10.83	10,19
	R^2^	0.9962	0.9467	0.9697	0.9633

**Table 4:** Thermodynamic parameters for the Cu(II), Zn(II), Mn(II), and Fe(III) complexes with IDHA sorbed on Agro^®^ Hydrogel.

T [K]	ΔG^o^ [kJ/mol]	ΔH^o^ [kJ/mol]	ΔS^o^ [kJ/mol K]	R^2^
Cu(II)-IDHA = 1:1				
293	−7.35	5.88	−12.3	1.0000
313	−8.26			
333	−9.16			
Zn(II)-IDHA = 1:1				
293	−9.02	2.62	−17.7	0.9999
313	−9.81			
333	−10.61			
Mn(II)-IDHA = 1:1				
293	−9.45	3.05	−14.8	1.0000
313	−10.30			
333	−11.15			
Fe(III)-IDHA = 1:1				
293	−8.40	4.44	−13.6	0.9999
313	−9.27			
333	−10.15			

**Table 5:** Desorption efficiency of Mn(II) from Agro^®^ Hydrogel using different desorbing agents.

Desorbing Agent	Desorption Efficiency %
0.1 M HNO_3_	98.0
0.1 M HCl	98.4
1 M HNO_3_	100.0
1 M HCl	100.0
distilled water	46.0

## Data Availability

The data presented in this study are available on request from the corresponding author.

## Supplementary Materials

The following supporting information can be downloaded at: https://www.mdpi.com/article/10.3390/ma17010141/s1.
