# Supplementary material for: Improved Soil Amendment by Integrating Metal Complexes and Biodegradable Complexing Agents in Superabsorbents
Source: Materials (Basel). 2023 Dec 27;17(1):141. doi: 10.3390/ma17010141 (PMC10779777; doi:10.3390/ma17010141)
Supplement: Supplementary file 1 [file materials-17-00141-s001.zip › materials-2780505-supplementary.pdf]

Improved soil amendment by integrating metal complexes and biodegradable complexing agents in superabsorbents

Alicja Drozd [1], Yongming Ju [2,3] and Dorota Kołodyńska [4,*]

[1] Analytical Department, Łukasiewicz Research Network—New Chemical Syntheses Institute, Al. Tysiąclecia Państwa Polskiego 13a, 24-110 Puławy, Poland; alicja.drozd@ins.lukasiewicz.gov.pl
[2] Nanjing Institute of Environmental Sciences, Ministry of Ecology and Environment (MEE),
Nanjing 510655, China; juyongming@scies.org
[3] Innovative Laboratory for Environmental Functional Materials and Environmental Applications of Microwave Irradiation, South China Subcenter of State Environmental Dioxin Monitoring Center, South China Institute of Environmental Sciences, Ministry of Ecology and Environment (MEE),
Guangzhou 510655, China
[4] Faculty of Chemistry, Institute of Chemical Science, Department of Inorganic Chemistry, Maria Curie-Sklodowska University, M. Curie-Sklodowska Sq. 2, 20-031 Lublin, Poland
* Correspondence: dorota.kolodynska@mail.umcs.pl

Table S1. Physicochemical properties of Agro® Hydrogel.

**Properties**	**Agro® Hydrogel**
Appearance	white powder
Form	poly(acrylic-co-acrylamide) 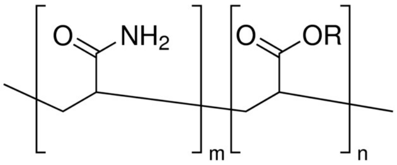 R=H or K
S_{BET} (m^2/g)	43
Water absorbency	380 g H_2O/g
Operating pH range	5-9
Bead size	0.300-1.000 mm<br>max 0.400 mm
Macroscan images	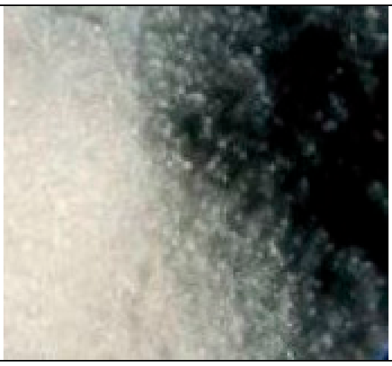
Macroscan images	

scans Agro® Hydrogel and Agro® Hydrogel@Cu-EDDS	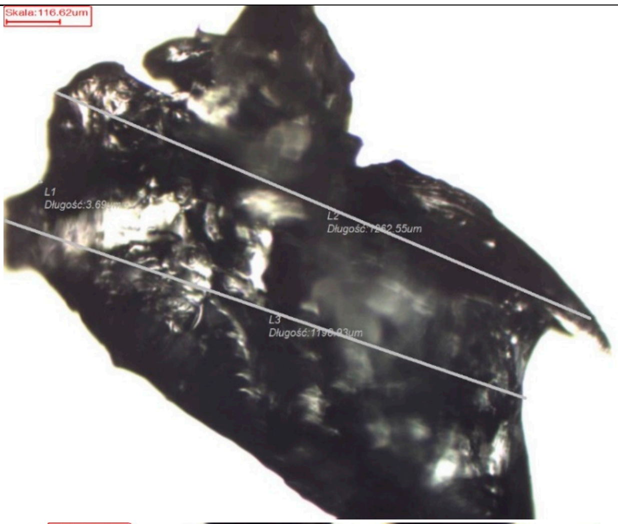 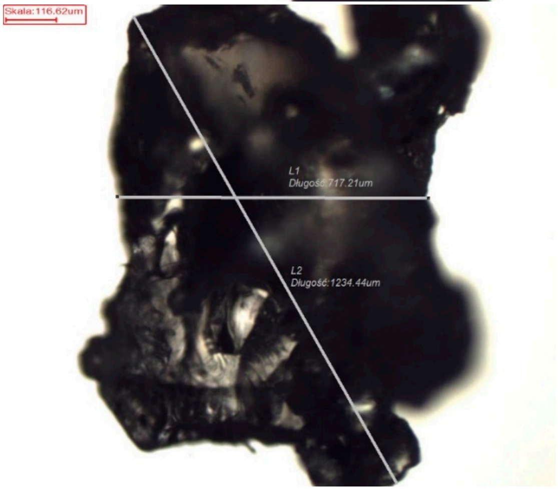

Table S2. The concentration of micronutrients used in the investigation and the abbreviations of the obtained complexes.

Salt	APCAs	Concentration	Abbreviation
$Cu(NO_3)_2{\cdot}3H_2O$	IDHA<br>EDDS<br>GLDA	$1x10^{-3}$ mol/L	Agro® Hydrogel@Cu-IDHA (A®H@Cu-IDHA)<br>Agro® Hydrogel@Cu-EDDS (A®H@Cu-EDDS)<br>Agro® Hydrogel@Cu-GLDA (A®H@Cu-GLDA)
$Zn(NO_3)_2{\cdot}6H_2O$	IDHA<br>EDDS<br>GLDA	$1x10^{-3}$ mol/L	Agro® Hydrogel@Zn-IDHA (A®H@Zn-IDHA)<br>Agro® Hydrogel@ Zn-EDDS (A®H@Zn-EDDS)<br>Agro® Hydrogel@ Zn-GLDA (A®H@Zn-GLDA)
$Mn(NO_3)_2{\cdot}H_2O$	IDHA	$1x10^{-3}$ mol/L	Agro® Hydrogel@Mn-IDHA (A®H@Mn-IDHA)

	EDDS<br>GLDA		Agro® Hydrogel@Mn-EDDS (A®H@Mn-EDDS)<br>Agro® Hydrogel@Mn-GLDA (A®H@Mn-GLDA)
$Fe(NO_3)_3{\cdot}9H_2O$	IDHA<br>EDDS<br>GLDA	1×10^{-3} mol/L	Agro® Hydrogel@Fe-IDHA (A®H@Fe-IDHA)<br>Agro® Hydrogel@Fe-EDDS (A®H@Fe-EDDS)<br>Agro® Hydrogel@Fe-GLDA (A®H@Fe-GLDA)

Calculations

The dried Agro® Hydrogel was stirred in distilled water for 24 h at 293 K. After filtration the insoluble parts were dried and weighed. G% was determined as:

$$G\% = (m_d - m_s) \times 100\% \quad (1)$$

where: m_d is the weight of dried Agro® Hydrogel and m_s is the swollen weight of Agro® Hydrogel.

As for the moisture retention (*M%*), the Agro® Hydrogel particles of 2 mm thickness were allowed to swell in water over night. Then they were placed in the petri dish at room temperature. The samples were weighed at the initial (m_o) and different time (m_t) intervals. The moisture retention capability (*M%*) was measured using *Eq.2*:

$$M\% = \frac{m_t}{m_0} \times 100\% \quad (2)$$

where: m_t is the weight of dried Agro® Hydrogel at the time *t* and m_o the weight of dried hydrogel at the initial time.

For the water/NaCl absorbency Agro® Hydrogel was immersed in distilled water or NaCl solution at room temperature. After proper time intervals the samples were separated by filtration. The water (Q_{H2O},%) and NaCl solution (Q_{NaCl},%) absorbencies of Agro® Hydrogel were determined by weighing the swollen samples. There were used *Eqs.3* and *4*, respectively [1,2]:

$$Q_{H_2O} = \frac{m_s - m_d}{m_d} \times 100\% \quad (3)$$

$$Q_{NaCl} = \frac{m_s - m_d}{m_d} \times 100\% \quad (4)$$

where: m_d and m_s are the weights of the dry Agro® Hydrogel and the water-swollen hydrogel, respectively. All the experiments were conducted three times to obtain the average values. Q_{H2O} was calculated as grams of water per a gram of sample.

The effect of the pH on the swelling was also verified in buffer solutions (pH 2, 4.01, 7 and 11). The procedures were the same as described above.

The sorption percentage (*%S*) and the amount of Cu(II), Zn(II), Mn(III) and Fe(III) complexes with IDHA, EDDS and GLDA adsorbed on the selected hydrogels (mg/g) were estimated using *Eqs.5* and *6*, respectively:

$$\%S = \frac{(C_0 - C_t)}{C_0} \times 100\% \quad (5)$$

$$q_t = (C_0 - C_t) \times \frac{V}{m} \quad (6)$$

where: the starting and final concentrations of M(II/III) complexes with IDHA, EDDS and GLDA solution (mg/L or M) are C_0 is C_t, V the volume of the solution (L) and m_d the mass of the dried hydrogels (g). Using C_e instead of C_t, the amounts of the metal complexes adsorbed at equilibrium were computed analogously.

The two adsorption isotherm models, Langmuir and Freundlich, were used to analyze the equilibrium adsorption data in order to evaluate the adsorption isotherm of IDHA, EDDS and GLDA complexes on polyacrylic superabsorbents. and they are expressed as *Eqs.7* and *8*, respectively [3], [4]:

$$\frac{1}{q_e} = \frac{1}{K_L q_0 C_e} + \frac{1}{q_0} \quad (7)$$

$$log q_e = \frac{1}{n} log C_e + log K_F \quad (8)$$

where: q_0 is the Langmuir monolayer adsorption capacity (mg/g), K_L is the Langmuir constant related to the free energy of the adsorption (L/mg), q_e is the amount of M(II) or M(III) complexes with IDHA, EDDS and GLDA sorbed at equilibrium (mg/ g), C_e is

the equilibrium concentration (mg/L), K_F is the Freundlich adsorption capacity (mg/g) and 1/*n* is the Freundlich constant related to the surface heterogeneity.

Another model is the Temkin isotherm which assumes that the heat of adsorption decreases linearly with the increase in coverage of the adsorbent. The linear form of the Temkin isotherm is presented in *Eq.9:*

$$q_e = BlnA + BlnC_e \quad (9)$$

where: *B*=(*RT b*) (*b* is the Temkin constant related to the heat of adsorption), *A* is the equilibrium binding constant, *R* is the constant (8.314 J/mol K) and *T* is the temperature (K). The values of *A* and *B* were obtained by plotting q_e vs. lnC_e and calculating the slope and intercept, respectively.

The Dubinin-Radushevich (D-R) isotherm model confirms the adsorption on both homogeneous and heterogeneous surfaces [5]. The linear form of the D-R isotherm is expressed mathematically as:

$$lnq_e = lnX_m - \beta_{\varepsilon^2} \quad (10)$$

where: X_m is the maximum adsorption capacity (mg/g) and

$$\varepsilon = RTln(1 + 1/C_e) \quad (11)$$

where: *R* is the constant (8.314 J/mol K), *T* is the temperature (K) and C_e is the equilibrium concentration of the adsorbate (mg/L).

The Gibbs free energy (ΔG°) was defined from *Eq.12*:

$$\Delta G° = RT\ln(K_C) \quad (12)$$

where: *R* is the constant (8.314 J/ mol K), *T* is temperature (K) and K_C is the equilibrium constant. (Δ*H*°) and (Δ*S*°) were calculated from the slope and intercept of the van’t Hoff plots of lnK_C vs. *1/T*.

The pseudo first order (PFO) and pseudo second order kinetic (PSO) models are utilized for the analysis of sorption data. The pseudo first order kinetic equation can be written as follows (*Eq.13*) [6]:

$$\log(q_e - q_t) = logq_{e-}\frac{k_1 t}{2.303} \qquad (13)$$

where: q_e and q_t denote the amounts of adsorption at equilibrium and time t (mg/g) respectively; k_1 is the rate constant of the pseudo first order adsorption (1/min). Based on the plot of $log(q_{e,1}$-$q_t)$ vs. t the kinetic parameters were calculated.

The pseudo second order model is expressed as (*Eq.14*):

$$\frac{t}{q_t} = \frac{t}{q_e} + \frac{1}{k_2 q_e^2} \qquad (14)$$

where: q_e and q_t denote the amounts of adsorption at equilibrium and time t (mg/g) respectively, k_2 is the rate constant of the pseudo second order adsorption (g/mg min). The kinetic parameters were calculated based on the plots of t/q_t vs. t.

Another kinetic equation used for the analysis of the experimental data is the Weber-Morris equation, i.e. the intraparticle diffusion model (IPD) given below:

$$q_t = k_i t^{1/2} + C \qquad (15)$$

where: k_i is the intraparticle diffusion rate constant (mg/ g $min^{0.5}$), C is the intercept which reflects the boundary layer effect. The kinetic parameters were calculated based on the plots of q_t *vs.* $t^{0.5}$.

References

[1] Y. Masuda, T. Tanaka, T. Nakanishi, Ion-specific swelling behavior of poly(vinyl alcohol) gel prepared by γ-ray irradiation, Colloid Polym. Sci. 279 (2001) 1241–1244. https://doi.org/10.1007/s003960100556.

[2] X. Wang, H. Hou, Y. Li, Y. Wang, C. Hao, C. Ge, A novel semi-IPN hydrogel: Preparation, swelling properties and adsorption studies of Co (II), J. Ind. Eng.

Chem. 41 (2016) 82–90. https://doi.org/10.1016/j.jiec.2016.07.012.

[3] N. Ahmad, F. Suryani Arsyad, I. Royani, A. Lesbani, Adsorption of methylene blue on magnetite humic acid: Kinetic, isotherm, thermodynamic, and regeneration studies, Results Chem. 4 (2022) 100629. https://doi.org/10.1016/j.rechem.2022.100629.

[4] F. Parsadoust, M. Shirvani, H. Shariatmadari, M. Dinari, Effects of GLDA, MGDA, and EDTA chelating ligands on Pb sorption by montmorillonite, Geoderma. 366 (2020) 114229. https://doi.org/10.1016/j.geoderma.2020.114229.

[5] T. Yousefi, M. Torab-Mostaedi, M.A. Moosavian, H.G. Mobtaker, Potential application of a nanocomposite:HCNFe@polymer for effective removal of Cs (I) from nuclear waste, Prog. Nucl. Energy. 85 (2015) 631–639. https://doi.org/10.1016/j.pnucene.2015.08.006.

[6] E.D. Revellame, D.L. Fortela, W. Sharp, R. Hernandez, M.E. Zappi, Adsorption kinetic modeling using pseudo-first order and pseudo-second order rate laws: A review, Clean. Eng. Technol. 1 (2020) 100032. https://doi.org/10.1016/j.clet.2020.100032.
